# Postoperative Patellar Height After Undergoing Total Knee Arthroplasty: Mechanical Axis Versus Kinematic Axis

**DOI:** 10.7759/cureus.24341

**Published:** 2022-04-21

**Authors:** Seleem Elkadi, Emily Krisanda, Brian J Panish, Stiles Donaldson, Eliana Schaefer, Malaak Hamzeh, John Bovill, Natasha Freed, Zachariah Elkordy, Seif El Masry, Gina Cach, Evan Jacquez, Evan Argintar

**Affiliations:** 1 Orthopaedic Surgery, Georgetown University School of Medicine, Washington, DC, USA; 2 Medical School, Georgetown University School of Medicine, Washington, DC, USA; 3 Orthopaedic Surgery, Georgetown University Hospital, Washington, DC, USA; 4 Orthopaedic Surgery, Washington Hospital Center, Washington, DC, USA

**Keywords:** blackburne-peel index, caton-deschamps index, insall-salvati index, patellar height, mechanical axis, kinematic axis, total knee arthroplasty

## Abstract

Introduction

When performing total knee arthroplasty (TKA), surgeons may use either the mechanical alignment (MA) or the kinematic alignment (KA) to guide implant placement and joint balancing. By measuring preoperative and postoperative patellar height (PH), surgeons can predict knee stability after TKA. Improper PH is associated with knee instability which may complicate the postoperative course and lead to patient dissatisfaction or need for revision. The purpose of this study is to measure patellar height using the Insall-Salvati Index (ISI), Caton-Deschamps Index (CDI), and Blackburne-Peel Index (BPI) preoperatively and postoperatively in patients who underwent TKA with either MA or KA to assess for changes in patellar height.

Methods

We performed a retrospective eight-year review of 256 patients who underwent TKA with either MA or KA by a single surgeon at a single hospital site. We obtained demographic data, including gender, age, and BMI, via the electronic health record. Furthermore, we calculated the ISI, CDI, and BPI using necessary parameters from preoperative and postoperative radiographs. We used these measurements to assess any statistically significant difference in postoperative PH.

Results

The MA cohort consisted of 104 patients with an average age of 63 years and an average BMI of 34.1 kg/m^2^. The KA cohort included 152 patients with an average age of 64 years and an average BMI of 34.9 kg/m^2^.

For the MA population, the average postoperative score with ISI was 1.10 [1.05 to 1.16] (p < 0.001), with CDI was 1.05 [0.98 to 1.11] (p < 0.001), and with BPI was was 0.94 [0.89 to 0.99] (p < 0.001). While for the KA population, the average postoperative score with ISI was 1.03 [0.99 to 1.06] (p = 0.17), with CDI was 0.87 [0.82 to 0.91] (p = 0.15), and with BPI was 0.82 [0.78 to 0.86] (p = 0.34).

Conclusion

TKA with a KA has a statistically significant improvement in postoperative PH and better postoperative maintenance of preoperative PH. Improved PH may lead to increased patellofemoral stability and superior postoperative outcomes in patients undergoing TKA. Future studies should focus on whether differences in preoperative and postoperative PH measurements result in changes in clinical outcomes in patients with MA versus KA TKA.

## Introduction

Total knee arthroplasty (TKA) involves resurfacing the femur to fit a prosthesis that replicates the distal femur’s shape and resurfacing the proximal tibia to hold a polyethylene bearing while preserving the collateral ligaments. The goal of TKA is pain reduction and improvement of knee functionality. TKA is a reliable treatment option for individuals suffering from knee pain secondary to arthritis and deformity that failed conservative treatment, including weight reduction, NSAIDs, activity modification, bracing, and physical therapy [1]. The goal of TKA is to improve patient quality of life (QOL), but 20% of patients report post-operative dissatisfaction [2]. 

There are various alignment strategies for TKA, including mechanical alignment (MA) and kinematic alignment (KA). MA strives to restore normal alignment by creating a neutral alignment or slight valgus to the knee, improving patient function and long-term implant survival [3-5]. The mechanical axis is a line drawn from the center of the femoral head to the center of the ankle joint. The cuts performed during TKA are perpendicular to this line. The mechanical axis divides into the femoral mechanical axis, from the head of the femur to the intercondylar notch of the distal femur, and the tibial mechanical axis, from the center of the proximal tibia to the center of the ankle [3,6]. Studies have shown that the MA method results in kinematic consequences by changing the angle and level of the distal femoral, posterior femoral, and tibial joint lines [7]. In addition, other studies have shown that a portion of the population has a natural varus alignment to the knee rather than a neutral alignment [3,7]. It has also been suggested that the MA method causes ligament imbalance, which is a possible explanation for increased rates of patient dissatisfaction after TKA [8].

Kinematic alignment (KA) is an alternative technique that differs from MA by considering the relationship between the femur, patella, and tibia rather than the center of the femoral head or ankle [9]. KA aims to restore pre-arthritic knee function by aligning the three kinematic axes: the femoral transverse axis, the tibial transverse axis, and the longitudinal axis. The tibia flexes and extends about the femoral transverse axis. The patella flexes and extends about the tibial transverse axis, located anterior, posterior, and parallel to the femoral transverse axis. The tibia internally and externally rotates on the femur about the longitudinal axis of the tibia, located perpendicular to the first two axes [9]. The cut done during TKA is on the longitudinal axis. These axes mimic the dynamic motion of the knee. 

There is no consensus on whether restoring physiologic alignment in KA versus neutral alignment in MA leads to improved outcomes and patient satisfaction [3,8]. KA may result in early failure of the prosthesis due to a varus alignment [4]. However, other studies show that kinematic alignment with varus does not affect implant survival or function, making it a viable alternative to MA [7]. There is no current consensus on whether the goal for alignment should be neutral, varus, valgus, or with the restoration of native alignment [4,7].

Postoperative patellar height measurements can predict knee stability after TKA. TKA commonly alters patellar height, resulting in suboptimal functional outcomes postoperatively [10]. Malalignment of the patella may cause instability, pain, and decreased range of motion (ROM) [10-14]. Common complications are patella alta, superior patella displacement, and patella baja, inferior patella displacement. Evaluation of postoperative patellar height (PH) can assess a more precise restoration of anatomic patellar positioning, optimizing results in TKA. Patellar height can be measured using three validated indices: Insall-Salvati Index (ISI), the Caton-Deschamps Index (CDI), and Blackburn-Peel Index (BPI) [5, 15-16]. 

ISI is the ratio comparing the diagonal length of the patella to the length of the patellar tendon. Normal values lie between 0.8 and 1.2 [5]. CDI is the ratio comparing the distance from the distal portion of the articular surface of the patella to the anterosuperior angle of the tibial plateau (AT) to the length of the articular surface of the patella (AP). Normal values lie between 0.6 and 1.2 [16]. BPI is the ratio comparing the articular length of the patella to the height of the lower pole of the articular cartilage above the tibial plateau. Normal values lie between 0.54 and 1.06 [15].

Currently, there is a paucity of literature examining whether MA or KA TKA results in a more accurate restoration of PH. This study measured ISI, CDI, and BPI both preoperatively and postoperatively for patients who underwent TKA with either the MA or KA method to examine which technique established a more exact recreation of PH relative to the patient’s native anatomy.

## Materials and methods

Data collection

The Institutional Review Board at the Georgetown University School of Medicine approved this retrospective study. Using the electronic medical record, we gathered a list of all patients who underwent primary TKA performed by the same physician at one hospital from January 2013 to December 2020. We reviewed patient charts meeting the criteria to ensure adequate preoperative and postoperative imaging was present in the network-wide picture archiving and communication system (PACS). All patients had patellar resurfacing done. Charts without adequate imaging for review were excluded. 

After identifying the patients who met inclusion criteria, we obtained demographic data, including height, weight, BMI, age at surgery, gender, the axis used (mechanical vs. kinematic), and laterality (right or left). Next, three investigators used preoperative and postoperative imaging to obtain the ISI, CDI, and BPI. The imaging software used was Visage 7 (Visage Imaging Inc., San Diego, CA). Using the measuring tool at the original magnification (100%), we measured each individual length of the indices. A separate investigator reassessed all the images for accurate measurements. Then, we calculated the ISI, CDI, and BPI for each patient preoperatively and postoperatively. 

Statistical analysis of demographic data

Next, we assessed the demographic data for both the MA and KA populations for mean, standard deviation, ranges of the ages, BMI, and gender distribution. We compared the age and BMI between the two populations using either a two-sample equal variance t-test or a two-sample unequal variance t-test depending on the f-test for the equality of variances. Finally, we used Pearson’s chi-squared test to compare both populations using the MA population as the expected value.

Statistical analysis of postoperative data

We calculated the mean and confidence intervals of the postoperative values for both populations for the postoperative data. We used a two-sample t-test with unequal variances to compare the average postoperative data with the ideal value. All confidence intervals used in this study were 95% confidence intervals. Next, we used either a two-sample equal variance t-test or a two-sample unequal variance t-test depending on the result of the f-test to compare the postoperative data between the two populations.

Statistical analysis of the difference between the preoperative and postoperative data

To compare the preoperative data with the postoperative data, we first calculated the absolute value of the difference between both the preoperative ratio and ideal value and the postoperative ratio and the ideal value. Next, we divided these differences by the ideal value and multiplied by 100% to produce the relative percent the measured value deviated from the ideal value. Finally, we subtracted the relative preoperative value from the relative postoperative value. A positive value signifies improved PH after the TKA, while a negative value signifies worsened PH. We used a value of 1.0 for the ISI, 0.9 for CDI, and 0.8 for BPI for the ideal values.

Finally, we calculated the averages and confidence intervals of the relative difference and performed either a two-sample equal variance t-test or a two-sample unequal variance t-test depending on the f-test for the equality of variances.

## Results

Demographic data

The study included 256 patients undergoing TKA. The mechanically aligned group consisted of 104 patients, 27 male and 77 female. The average age was 63 years, ranging from 43-85 years, and an SD of 9.66 years. The average BMI was 34.1 kg/m2, range 20.7-57.2 kg/m2, with an SD of 7.37 kg/m2. 

The kinematically aligned group included 152 patients, 47 male and 105 female. The average age was 64 years, ranging from 43-95 years, and an SD of 9.46 years. The average BMI was 34.9 kg/m2, ranging from 21.0 to 57.6 kg/m2, with an SD of 6.76 kg/m2. 

A chi-squared test showed no significant difference in gender between the MA and KA population (p = 0.124). The f-test for age revealed equivalent variances between populations (p = 0.80). Subsequently, a two-sample equal variance t-test showed no significant difference in age between groups (p = 0.546). The f-test for BMI revealed no significant difference in variance between groups (p = 0.33), and the two-sample equal variance t-test revealed no significant difference between populations (0.380) (Table 1).

**Table 1:** Patient Demographics. BMI = Body-Mass Index

	Mechanical	Kinematic	p-value
Sample size (n)	104	152	
Age (years) (SD)	63 (9.7) (range 43 to 85)	`64 (9.5) (range 43 to 95)	0.546
Sex			0.124
Male	27 (26%)	47 (31%)	
Female	77 (74%)	105 (69%)	
BMI (SD)	34.1 (7.4) (range 20.7 to 57.2)	34.9 (6.8) (range 21.0 to 57.5)	0.380

Postoperative data

For the MA population, the average postoperative score with ISI was 1.10 [1.05 to 1.16] (p < 0.001), while for the KA population, the average was 1.03 [0.99 to 1.06] (p = 0.17). The f-test revealed a statistically significant difference in variance between groups (p = 0.04). A two-sample unequal variance t-test revealed a statistically significant difference in variance between the average postoperative score for ISI in MA versus KA (p = 0.018). 

For the MA population, the average postoperative score with CDI was 1.05 [0.98 to 1.11] (p < 0.001), while for the KA population, the average was 0.87 [0.82 to 0.91] (p = 0.15). The f-test showed a statistically significant difference in variance between groups (p = 0.01). A two-sample unequal variance t-test revealed a statistically significant difference between the average postoperative score for CDI in MA versus KA (p < 0.001).

For the MA population, the average postoperative score with BPI was 0.94 [0.89 to 0.99] (p < 0.001), while for the KA population, the average score was 0.82 [0.78 to 0.86] (p = 0.34). The f-test revealed no statistically significant difference in variance between groups (p = 0.96). A two-sample equal variance t-test revealed a statistically significant difference between the average postoperative score for CDI in MA versus KA (p < 0.001) (Table 2).

**Table 2:** Patellar Height Postoperative scores: Mechanical vs. Kinematic. * = significant at p < 0.05 level. ISI = Insall-Salvati Index, CDI = Caton-Deschamps Index, BPI = Blackburn-Peel Index CI = Confidence Interval

	Mechanical		Kinematic		
	Average	95% CI	Average	95% CI	p-value
ISI	1.10	[1.05, 1.16]	1.03	[0.99, 1.06]	0.018*
CDI	1.05	[0.98, 1.11]	0.87	[0.83, 0.91]	< 0.001*
BPI	0.94	[0.89, 0.99]	0.82	[0.78, 0.86]	< 0.001*

Relative difference data

For the MA population, the average preoperative and postoperative difference with ISI was -4.56% [-8.23% to -0.88%] (p = 0.017), while for the KA population, the average difference was -1.94% [-4.27% to 0.39%] (p = 0.10). F-test for equality of variance revealed a statistically significant difference between the groups (p = 0.003). A two-sample unequal variance t-test showed no statistically significant difference between preoperative and postoperative scores for ISI in MA versus KA (p = 0.24).

For the MA population, the average preoperative and postoperative difference with CDI was -8.72% [-15.22% to -2.22%] (p = 0.01), while for the KA population, the average difference was 0.64% [-3.47% to 4.76%] (p = 0.76). The f-test revealed a difference in variance between groups (p = 0.003). A two-sample unequal variance t-test showed a statistically significant difference in preoperative and postoperative scores for ISI in MA versus KA (p = 0.02).

For the MA population, the average preoperative and postoperative BPI difference was -7.38% [-13.70% to -1.06%] (p = 0.02), while the average difference for the KA population was -3.62% [-8.12% to 0.89%] (p = 0.12). The f-test showed equal variance between groups (p = 0.10). A two-sample unequal variance t-test showed no statistically significant difference between preoperative and postoperative scores for BPI in MA versus KA (p = 0.33) (Table 3).

**Table 3:** Maintenance of Patellar Height Comparison: Mechanical vs. Kinematic. * = significant at p < 0.05 level. * = significant at p < 0.05 level ISI = Insall-Salvati Index, CDI = Caton-Deschamps Index, BPI = Blackburn-Peel Index CI = confidence interval

	Mechanical		Kinematic		
	Average	95% CI	Average	95% CI	p-value
ISI	-4.6	[-8.2, -0.9]	-1.9	[-4.3, 0.4]	0.240
CDI	-8.7	[-15.2, -2.2]	0.6	[-3.5, 4.8]	0.018*
BPI	-7.4	[-13.7, -1.1]	-3.6	[-8.1, 0.9]	0.330

## Discussion

While TKA is a reliable treatment option for patients with osteoarthritis of the knee refractory to more conservative measures, 20% of patients report dissatisfaction postoperatively [2]. Using a kinematic alignment instead of a mechanical alignment for operative planning could lead to higher patient satisfaction rates and overall implant stability. Theoretically, the kinematic alignment aims to restore pre-arthritic knee function, promoting a return to optimal knee function. TKA often leads to alterations in patellar height, which can produce complications such as instability, pain, and decreased ROM. Therefore, assessing proper surgical restoration or maintenance of patellar height could shed light on whether there is a difference in outcomes between MA and KA TKA. As KA aims to align the three kinematic axes, we hypothesize that TKA performed with KA will better restore PH than TKA done with MA.

To compare the MA and KA populations accurately, we ensured that the populations were statistically equal. Therefore, we performed a chi-squared test to assess for a possible difference in gender between the MA and KA populations and t-tests to assess for a difference in age or BMI between the two groups, determining a p-value of 0.124 for gender, 0.546 for age, and 0.380 for BMI. We determined that these populations were sufficiently similar for comparison based on the demographic data. 

Across all three indices, the patients who had undergone TKA using the KA method had a statistically significant improvement in postoperative PH compared to patients who had undergone TKA with the MA method. Therefore, TKA with KA leads to a more precise PH restoration than a TKA done with MA. 

To assess for maintenance of preoperative PH following surgery, we compared preoperative PH with postoperative PH by subtracting the preoperative and postoperative values of each index from the ideal value. We took the absolute value because we only wanted to know the distance between the measured and ideal value. Next, the distance was divided by the ideal value and multiplied by 100 to obtain the percentage of the distance the measured value deviated from the ideal value. Then, we subtracted the preoperative percentage by the postoperative percentage to assess whether the distance from the ideal value changed postoperatively. A positive value indicated an improvement in PH postoperatively. 

After performing this calculation, we found a statistically significant derangement of PH in the patients who had undergone TKA with a MA compared to preoperative calculations across all three indices. However, the patients who had undergone TKA with KA had no significant difference in PH compared to preoperative calculations across all three indices. Therefore, we determined that TKA with KA maintains preoperative PH while TKA with MA does not. 

When comparing this difference across the MA and KA populations, we saw a statistically significant decrease in the difference in PH in the KA population based on the CDI. The other two indices did not show a statistically significant difference. This data comparison between the two populations provides more evidence that the KA axis results in better postoperative PH preservation than the MA axis.

Maintaining the preoperative PH could be critical in both short-term and long-term outcomes from TKA. Alterations in PH are related to instability, pain, and decreased knee ROM. Ensuring the best possible postoperative PH may increase knee stability and function, improving patient satisfaction. Furthermore, the knee’s soft tissue adjusts to the chronic changes associated with osteoarthritis, and maintaining preoperative PH could lead to fewer short-term adjustments by the body. Additionally, since KA aims to restore pre-arthritic knee function, patients may see long-term benefits as their knee heals to the pre-arthritic state. Future studies should focus on short-term and long-term clinical outcomes based on knee stability, pain, ROM, and function.

Limitations

The retrospective nature of this study limits the power of the results. Furthermore, there is a risk of human error with any data, including measurements. There was an attempt to mitigate this by having a single investigator review all the measurements. Another limitation is all the patients were operated on by a single surgeon at a single facility. Surgeon familiarity with the method and available resources may impact TKA outcome. Future studies should aim to have a prospective or randomized trial and have a standardized or computerized method to measure patellar height.

## Conclusions

This study has provided evidence that performing total knee arthroplasty with reference to the kinematic axis rather than the mechanical axis of the knee leads to a better postoperative PH and perhaps maintenance of preoperative PH, which could play a role in improving patient satisfaction with their TKA. Future studies should aim to be either prospective or randomized and assess short-term and long-term satisfaction in patients with knee function. These studies would improve understanding of whether the kinematic axis method of total knee arthroplasty leads to improved patient outcomes.
